# Diagnostic value of cellular inflammatory markers in CNS tumor patients undergoing glucocorticoid therapy

**DOI:** 10.3389/fonc.2026.1764339

**Published:** 2026-02-18

**Authors:** Sofia Sklyar, Daria Sitovskaya, Nataliya Dryagina, Alexei Ulitin, Victor Olyushin, Stephanie E. Combs, Konstantin Samochernykh, Maxim Shevtsov

**Affiliations:** 1 Branch of Almazov National Medical Research Centre, Polenov Neurosurgical Institute, Saint-Petersburg, Russia; 2 Department of Neurosurgery, Institute of Medical Education Almazov National Medical Research Centre, Saint-Petersburg, Russia; 3 Department of Radiation Oncology, Technische Universität München (TUM), Munich, Germany; 4 Laboratory of Biomedical Nanotechnologies, Institute of Cytology of the Russian Academy of Sciences (RAS), Saint Petersburg, Russia

**Keywords:** glioblastoma, immune response, lymphocyte-to-monocyte, meningioma, metastatic CNS lesion, neutrophil-to-lymphocyte ratio, pan-immune-inflammation value

## Abstract

**Introduction:**

While inflammation is recognized as a key driver of solid tumor progression, the diagnostic and pathophysiological relevance of cellular inflammatory biomarkers in central nervous system (CNS) neoplasms remains incompletely defined.

**Materials and methods:**

Clinical and hematological data were analyzed from 44 patients with glioblastoma, 32 with brain metastases, and 33 patients with meningioma who underwent primary surgical treatment between 2024 and 2025. A control group of healthy age-matched volunteers (n = 20) was included for comparison. The diagnostic performance of cellular inflammatory markers was evaluated across tumor entities, with additional stratification according to glucocorticosteroid exposure.

**Results:**

Patients with intracerebral tumors exhibited higher neutrophil and monocyte counts, as well as elevated neutrophil-to-lymphocyte ratio (NLR) and pan-immune inflammation value (PIV), along with a lower lymphocyte-to-monocyte ratio (LMR) compared to both the meningioma group and healthy volunteers (p<0.05). In glioblastoma patients, dexamethasone administration significantly affected neutrophil and monocyte counts, NLR and PIV (p<0.05). In the group with metastatic brain lesions, glucocorticoid therapy led to an increase in neutrophil count and NLR (p<0.05). The LMR level was not influenced by dexamethasone.

**Conclusion:**

For patients with glioblastoma, the LMR serves as a diagnostically significant inflammatory biomarker independent of glucocorticoid administration. In patients with metastatic brain lesions, the most significant diagnostic value was demonstrated by monocyte counts, PIV and LMR.

## 1 Introduction

Inflammation is recognized as a key hallmark of oncogenesis (1–3). This process, initially protective, becomes chronic in the tumor context, characterized by a restructuring of the tumor microenvironment (TME), accumulation of immunosuppressive cells, and suppression of effector immune responses (1, 2). This ultimately promotes active tumor proliferation, invasion, angiogenesis, and metastasis (1, 4, 5). It is hypothesized that the restructuring of the immune system within the TME induces changes in systemic inflammatory responses, reflected by shifts in the quantities of circulating immune cells, metabolites, and enzymes.

Numerous studies have demonstrated the high prognostic potential of inflammatory markers in various cancers (6–9). In routine clinical practice, cellular inflammation indices such as the neutrophil-to-lymphocyte ratio (NLR), lymphocyte-to-monocyte ratio (LMR), platelet-to-lymphocyte ratio (PLR), and the more recently introduced pan-immune inflammation value (PIV) are now widely used (6–9). These markers are characterized by ease of determination and calculation, low cost, and significant clinical utility.

Neutrophil-to-lymphocyte ratio (NLR), the most widely used cellular inflammation marker, is associated with both nonspecific (mediated by neutrophils) and adaptive immune responses, the latter involving active lymphocyte participation. Elevated NLR levels have been shown to be inversely associated with survival in patients with metastatic non-small cell lung cancer, bladder cancer, melanoma, and urothelial carcinoma (6, 10, 11).

Lymphocyte-to-monocyte ratio (LMR) is calculated by dividing the absolute lymphocyte count by the absolute monocyte count. In general oncology, a higher LMR has been established as a factor associated with better patient prognosis (6, 10, 11). Conversely, a decreased LMR may result from low lymphocyte counts – the main effectors of the adaptive immune response – and/or an elevated monocyte count. Monocytes play a dual role, and lymphopenia is recognized in oncology as a highly unfavorable prognostic factor (12).

Monocytes are currently among the most promising targets in immuno-oncology research. Following differentiation, these cells can either exert a protective function by eliminating pathological cells or contribute to oncogenesis by recruiting tumor-associated macrophages and fostering an immunosuppressive microenvironment (13, 14). Accordingly, high peripheral monocyte counts have been reported to predict poor survival in cancer patients.

The platelet-to-lymphocyte ratio (PLR) incorporates both lymphocyte and platelet levels. Platelets activated by the tumor microenvironment inhibit the interaction between tumor cells and cytotoxic immune cells and support tumor growth through the secretion of various factors. Consequently, thrombocytosis is associated with poor outcomes in cancer patients (15).

The pan-immune-inflammation value (PIV), a newer immune response biomarker, is emerging as a promising primary cellular inflammation marker for assessing prognosis and disease progression in oncology (3, 16, 17). PIV provides a comprehensive assessment by incorporating all major cellular players of the immune response. It is calculated as the product of the absolute neutrophil, monocyte, and platelet counts divided by the absolute lymphocyte count.

Fundamental and clinical studies over the past decade have refuted the concept of the central nervous system (CNS) as an immunologically isolated organ (14, 18–20). In the contemporary paradigm, the CNS is viewed as a structure with a unique, dynamic immune environment that is closely linked to the body’s systemic immune network and responds to the formation of pathological foci with specific inflammatory reactions (14, 18, 21). It has been established that cerebral neoplasms induce immune activation, leading to the migration of immune cells into the peritumoral zone, their subsequent reprogramming and the establishment of chronic neuroinflammation (14). Persistent immunosuppressive and neuroinflammatory processes, driven by the expression of various mediators and the presence of tumor-associated immune cells, lead to active proliferation and rapid migration of tumor cells, as well as resistance to administered therapy (22). It is important to emphasize that the common use of glucocorticoids to control peritumoral edema in CNS tumors exacerbates local immunosuppression (20).

Undoubtedly, the distinct origin, biology, and “behavior” of various cerebral tumors determine the specific characteristics of the local immunophenotype. Given the foregoing, it can be anticipated that systemic inflammatory responses in CNS tumors will exhibit a unique profile distinct from those in extracranial malignancies, and will also vary among different histological subtypes of brain neoplasms. Investigating these specific features offers promising prospects for introducing novel diagnostic and prognostic criteria into clinical neuro-oncological practice and for shaping new therapeutic approaches.

To date, only a limited number of studies have assessed the clinical significance of cellular inflammatory markers in patients with CNS tumors, with most focusing on malignant gliomas. Overall, the results have been highly contradictory. Some studies identify the NLR as the primary diagnostic and prognostic inflammatory biomarker, while others highlight LMR or PLR (23–27). It should be noted that several of these studies did not differentiate between histological subtypes and did not account for the use of GCS.

We therefore conducted an open-label single centre prospective study to assess the diagnostic value of the most widely used cellular inflammatory markers – NLR, LMR, PLR and PIV – in patients with tumors of the central nervous system. These included intraparenchymal brain tumors (glioblastoma and metastatic lesions) and an extracerebral tumor (meningioma). We specifically aimed to determine marker levels in the context of GCS use.

## 2 Materials and methods

### 2.1 Study population

This study included 109 adult patients (age >18 years) with newly diagnosed brain tumors who underwent surgical resection at the Almazov National Medical Research Center between 2024 and 2025. Study protocol was approved by the Ethics Committee of the Almazov Medical Research Centre (approval No. 2011-23 from 20.11.2023) and was conducted in accordance with the Declaration of Helsinki. Written informed consent was obtained from all participants. All studies were conducted in compliance with applicable guidelines and regulations.

The inclusion criteria were: (1) age over 18 years, and (2) presence of a supratentorial brain tumor confirmed by neuroimaging.

The exclusion criteria were as follows:

- Diagnosis of immunodeficiency or an active autoimmune disease;
- Presence of any malignancy outside the CNS at enrollment;
- History of prior radiation therapy, chemotherapy, or immunotherapy for a brain tumor;
- Presence of acute infectious or inflammatory diseases at the time of evaluation;
- Use of antibacterial, antiviral, or antifungal medications.

### 2.2 Blood sample analysis

Blood samples were collected from patients in a state of clinical stability. Stability was defined as the absence, for the preceding 72 hours, of any of the following: acute neurological deterioration, status epilepticus or a cluster of seizures, or signs of increased peritumoral edema requiring an escalation in dexamethasone dosage. The dexamethasone dose remained unchanged from its initiation until the time of blood sampling.

Venous blood samples were routinely collected two to three days prior to neurosurgical intervention for clinical and biochemical analysis, including C-reactive protein (CRP) measurement. Patients with CRP levels greater than 5 mg/L were excluded from the study. A complete blood count (CBC) with differential was performed on a Sysmex XN-550 hematology analyzer using Sysmex reagents and controls (Sysmex, Сhuo-ku, Japan). Biochemical analysis was conducted on an Integra 400 Plus analyzer (Roche Diagnostics, Indianapolis, USA). The laboratory parameters assessed included absolute neutrophil, lymphocyte, monocyte, and platelet counts. Inflammatory indices were calculated using established formulas:

1. NLR index (neutrophil-to-lymphocyte ratio): the ratio of the absolute number of neutrophils (10^9^/L) to the absolute number of lymphocytes (10^9^/L);
2. LMR index (lymphocyte-to-monocyte ratio): the ratio of the absolute number of lymphocytes (10^9^/L) to the absolute number of monocytes (10^9^/L);
3. PLR index (platelet-to-lymphocyte ratio): the ratio of the absolute number of platelets (10^9^/L) to the absolute number of lymphocytes (10^9^/L);
4. PIV index (Pan-immune-inflammation value): the ratio of the product of the absolute numbers of platelets (10^9^/L), neutrophils (10^9^/L), and monocytes (10^9^/L) to the absolute number of lymphocytes (10^9^/L);

To form a control group, a retrospective analysis of the medical records of healthy individuals undergoing annual medical examination at our medical institution was conducted. The control group excluded individuals with acute or chronic diseases in an active phase, a history of any oncological conditions, and those taking immunomodulatory, hormonal, or anti-inflammatory medications.

### 2.3 Histological analysis

The histological diagnosis was established by examining tumor tissue samples. The specimens were fixed in 10% buffered formalin, dehydrated through a standard ethanol series, and embedded in paraffin blocks. We examined histological sections stained with hematoxylin and eosin (H&E) and evaluated the results of immunohistochemical (IHC) staining. The following primary antibodies were used: anti-glial fibrillary acidic protein (Dako, USA; M0761) at 1:100; anti-IDH1R132H (Dianova, Germany; Dia-H09) at 1:50; anti-Ki-67 (Dako, USA; M7240) at 1:50; anti-pan-CK (Dako, USA) at 1:50; anti-SSTR2A (Abcam, USA; ab134152) at 1:300. The EnVision polymer detection system (Dako, USA) was used for visualization. Negative control reactions, omitting the primary antibodies, were performed to confirm staining specificity. Histological analysis and microphotography were conducted using a Leica Aperio AT2 scanning microscope and the Aperio ImageScope software (Leica Microsystems, Vista, USA). Results were interpreted according to the diagnostic criteria of the 5th edition of the World Health Organization Classification of Tumors of the Central Nervous System 2021 (28). Somatic mutations in the IDH1 and IDH2 genes (a panel of 17 mutations) were detected using real-time PCR with the IDH1/2 Mutation Detection Kit (EntroGen, USA; Cat. No. IDH-RT38).

### 2.4 Statistical analysis

The obtained data were processed using the SPSS Statistics (version 21.0) statistical program. Quantitative parameters were assessed for normal distribution using the Shapiro-Wilk test. Standard descriptive statistics, such as the median and the 25th and 75th percentiles of Me [Q1-Q3], were used to describe quantitative characteristics. Comparisons of two independent variables were performed using the Mann-Whitney test. When comparing more than two groups, the Kruskal-Wallis test was used. In the case of significant differences, paired comparisons were performed to identify distinct groups using the Mann-Whitney U test, all-versus-all, with multiple comparison error correction using the Benjamini-Hochberg method. ROC analysis was used to analyze the diagnostic significance of the parameters and assess threshold values. Based on this analysis, the area under the curve (AUC) was determined for test specificity and sensitivity. Results were considered statistically significant at p < 0.05.

## 3 Results

### 3.1 Patients’ characteristics

All patients were divided into three groups based on their histological diagnosis: glioblastoma (n=44), CNS metastases (n=32), and meningioma (n=33).

In the glioblastoma group, the median age at diagnosis was 62 years, with 24 (54.5%) women and 20 (45.5%) men. The median age in the metastasis group was 67 years, with 18 women (56.2%) and 14 men (43.8%). In the meningioma group, the median age was 63 years, with 25 (75.7%) women and 8 men (24.3%). The control group included 20 healthy volunteers with a median age of 63.5 years, 11 of whom were women (55%) and 9 men (45%). The clinical characteristics of the patients are presented in **Table 1**.

**Table 1:** Clinical and demographic characteristics of patients.

Parameters	Group 1 patients with glioblastoma	Group 2 patients with brain metastases	Group 3 patients with meningioma	Healthy controls
Number of patients	44	32	33	20
Average age	62 (57-68.5)	67 (62-75)	63 (58-69)	63.5 (57-67)
Women (%)	24 (54.5%)	18 (56.2%)	25 (75.5%)	11 (55%)
Men (%)	20 (45.5%)	14 (43.8%)	8 (24.3%)	9 (45%)
Dexamethasone prescription (number of patients, %)				
Yes	33 (75%)	24 (75%)	4 (12%)	0
No	11 (25%)	8 (25%)	29 (88%)	20 (100%)

All patients with brain metastases had a histologically verified primary tumor. Among the 32 cases, the primary tumor sites were as follows: cutaneous melanoma in 5 patients (15.6%), renal cell carcinoma in 3 (9.4%), and ovarian carcinoma in 3 (9.4%). Gastrointestinal tract cancers were less common, identified in only 2 patients (6.3%). The most frequent primary tumors were lung cancer (n=10, 31.3%) and breast carcinoma (n=9, 28.1%). All patients had previously received treatment for their primary malignancy, and the diagnosis of CNS metastases was established as a manifestation of disease progression.

Among patients with meningioma, 17 (51.5%) were diagnosed with World Health Organization (WHO) grade I tumors and 16 (48.5%) with WHO grade II tumors.

The use of preoperative GCS therapy was documented for each group. The dexamethasone dose ranged from 8 to 12 mg daily, tailored to the patient’s clinical presentation. Eligible patients were those who had received dexamethasone for a minimum of one week and up to three weeks prior to neurosurgical intervention. A majority of patients with glioblastoma (75%) and brain metastases (75%) received preoperative dexamethasone. In contrast, GCS were administered to only 4 patients (12.1%) in the meningioma group.

### 3.2 Comparison of preoperative inflammatory markers in patients with CNS tumors

Laboratory test results and inflammatory markers are summarized in **Table 2**.

**Table 2:** Preoperative laboratory parameters and assessed indices in three patient groups and group of healthy controls, Me [Q1-Q3].

Parameters	Group 1 patients with glioblastoma (n = 44)	Group 2 patients with brain metastases (n = 32)	Group 3 patients with meningioma (n = 33)	Group 4 healthy controls (n = 20)
laboratory indicators				
lymphocytes (x10^9^/L)	1.89 [1.18-3.05]	1.85 [1.17-2.46]	2.11 [1.43-2.61]	2.25 [1.84-2.73]
neutrophils (x10^9^/L)	8.36 [5.32-11.96] **α, β**	7.05 [5.11-8.14] **α, β**	3.54 [2.74-4.15]	3.39 [2.84-4.5]
monocytes (x10^9^/L)	0.72 [0.55-0.94] **α, β**	0.66 [0.46-0.85] **α, β**	0.49 [0.37-0.63]	0.48 [0.37-0.55]
platelets (x10^9^/L)	252 [185.5-282.25]	214 [185-276]	242 [10.5-300]	250 [233-278.25]
inflammatory blood markers				
NLR	4.29 [2.17-6.62] **α, β**	3.79 [2.89-5.6] **α, β**	1.6 [1.25-2.3]	1.7 [1.37-1.97]
LMR	2.6 [1.71-3.91] **α, β**	2.6 [1.88-3.77] **α, β**	4.2 [3.02-5.25]	4.55 [4.12-5.42]
PLR	130 [84.25-187.25]	122 [89-245]	121 [102-161.5]	105.5 [93.25-134.75]
PIV	759.5 [374-1433.94] **α, β**	641.43 [269.89-963] **α, β**	180.22 [132.71-361.31]	187 [142.5-250.75]

α p<0.05 vs meningioma.

β p<0.05 vs healthy controls.

NLR, neutrophil-to-lymphocyte ratio; LMR, lymphocyte-to-monocyte ratio; PLR, platelet-to-lymphocyte ratio; PIV, pan-immune-inflammation value.

First, it should be noted that no differences in the levels of the studied inflammatory markers were found between the glioblastoma and brain metastasis groups (p ≥ 0.05). Similarly, no significant differences were observed between the meningioma group and healthy volunteers (p ≥ 0.05).

Comparison across all four groups revealed no significant differences in lymphocyte and platelet counts and PLR.

**Figure 1:**
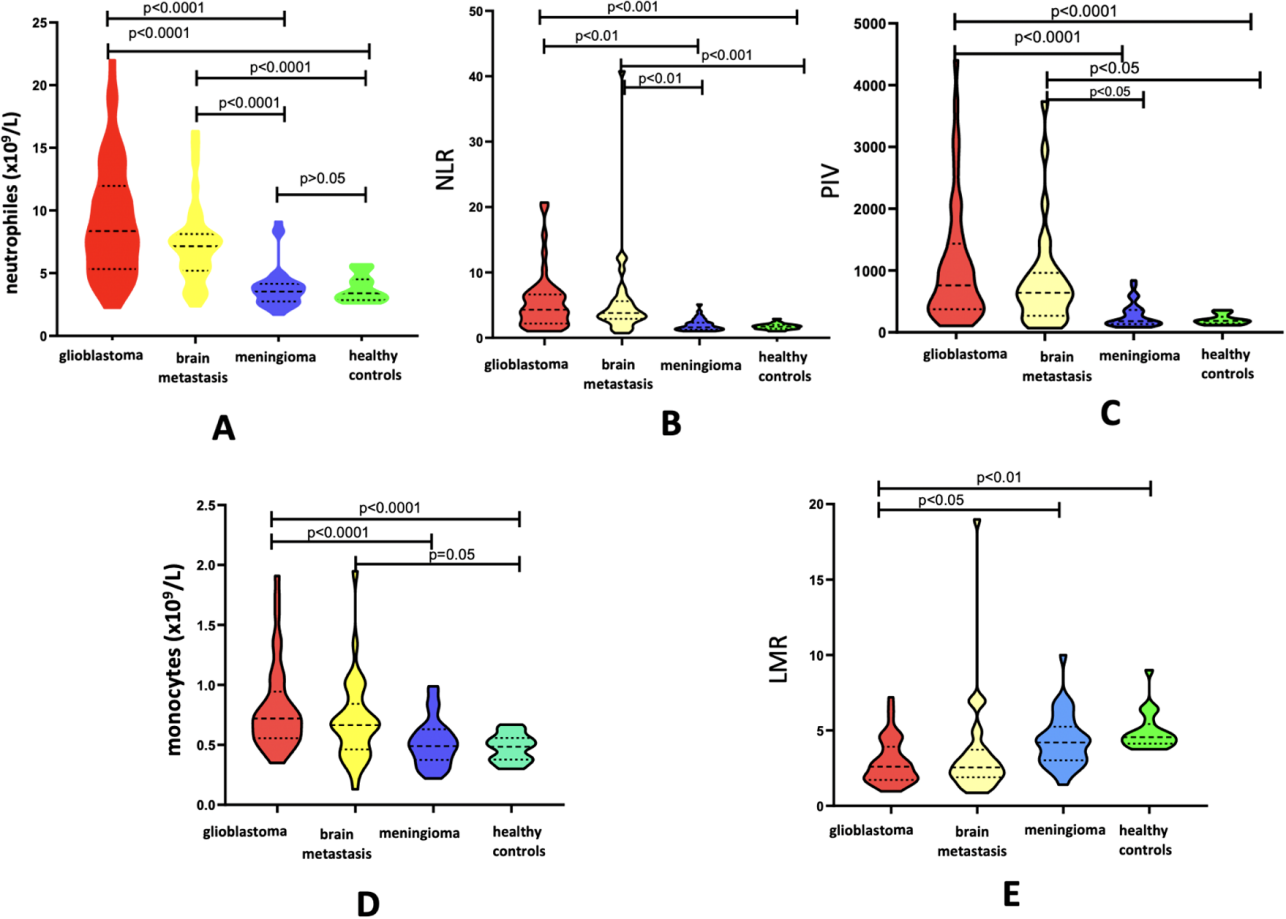
Violin diagram showing comparative results of preoperative inflammatory markers in glioblastoma, brain metastases, meningioma and healthy controls groups. **(A)** Neutrophils, **(B)** Monocytes, **(C)** NLR, **(D)** LMR, **(E)** PIV.

Patients with intracerebral CNS tumors (glioblastoma and brain metastases) exhibited statistically significantly higher levels of neutrophils [8.36 (5.32–11.96) and 7.05 (5.11–8.14), respectively], monocyte count [0.72 (0.55–0.94) and 0.66 (0.46-0.85), respectively], NLR [4.29 (2.17–6.62) and 3.79 (2.89–5.60)], and PIV [759.5 (374–1433.94) and 641.43 (269.89–963.00)] compared to patients with meningioma [3.54 (2.74–4.25), 0.49 (0.37-0.63), 1.60 (1.25–2.3), 180.22 (132.71–361.31)] and healthy volunteers [3.39 (2.84–4.5), 0.48 (0.37-0.55), 1.7 (1.37–1.97), 187.00 (142.50–250.75)] (**Figure 1**).

LMR values in the groups of patients with intracerebral CNS tumors (glioblastoma and brain metastases) [2.6 (1.71-3.91); 2.6 (1.88-3.77)] were significantly lower than in patients with meningioma [4.2 (3.02-5.25)] and healthy volunteers [4.55 (4.12-5.42)] (**Figure 1**).

### 3.3 Analysis of preoperative inflammatory markers in CNS tumor patients based on dexamethasone use

Furthermore, biomarker levels were analyzed in relation to preoperative GCS use among patients with glioblastoma and metastatic CNS lesions (**Tables 3**, **4**). Laboratory data for meningioma patients receiving GCS were not evaluated due to insufficient sample size for a meaningful comparison (4 vs. 29 patients not receiving steroids).

**Table 3:** Laboratory parameters in the glioblastoma group according to the administration of glucocorticoids, Me [Q1-Q3].

Parameters	Patients with glioblastoma		*P*-value
	With GCS (n=33)	Without GCS (n=11)	
lymphocytes (x10^9^/L)	1.92 [1.42-3.06]	1.26 [1.16 – 2.6]	p=0.437
neutrophils (x10^9^/L)	10.3 [7.56 -13.40]	4.54 [3.6-4.92]	p=0.0001
monocytes (x10^9^/L)	0.74 [0.61-1.06]	0.57 [0.52 -0.65]	p=0.015
platelets (x10^9^/L)	253 [187-288]	218 [195-268.5]	p=0.538
NLR	5.88 [3.20 -7.20]	2 [1.55 -4.19]	p=0.004
LMR	2.6 [1.7-3.66]	2.8 [1.97-4.14]	p=0.406
PLR	127 [85-182]	139 [82.5-186.5]	p=0.957
PIV	981 [450.25- 1647.7]	328.66 [150.7 – 527.2]	p=0.0001

GCS, Glucocorticoid therapy; NLR, neutrophil-to-lymphocyte ratio; LMR, lymphocyte-to-monocyte ratio; PLR, platelet-to-lymphocyte ratio; PIV, pan-immune-inflammation value.

**Table 4:** Laboratory parameters in the group of patients with brain metastases according to the administration of glucocorticoids, Me [Q1-Q3].

Parameters	Patients with brain metastases		*P*-value
	With GCS (n=24)	Without GCS (n=8)	
lymphocytes (x10^9^/L)	1.90 [1.31- 2.27]	1.57 [1.30 -2.22]	p=0.593
neutrophils (x10^9^/L)	7.64 [6.89-9.39]	3.93 [3.29-5.08]	p=0.0001
monocytes (x10^9^/L)	0.65 [0.47-0.92]	0.67 [0.41-0.70]	p=0.685
platelets (x10^9^/L)	214 [187-273]	226.5 [191-306.5]	p=0.815
NLR	4.4 [3.15-6.50]	2.93 [1.33-3.33]	p=0.013
LMR	2.6 [1.91-3.38]	2.4 [1.89-5.78]	p=0.717
PLR	120 [95-208.5]	174 [84-255]	p=0.623
PIV	650.82 [514.71-1112.58]	358.45 [181.36 -764.29]	p=0.091

GCS, Glucocorticoid therapy; NLR, neutrophil-to-lymphocyte ratio; LMR, lymphocyte-to-monocyte ratio; PLR, platelet-to-lymphocyte ratio; PIV, pan-immune-inflammation value.

**Figure 2:**
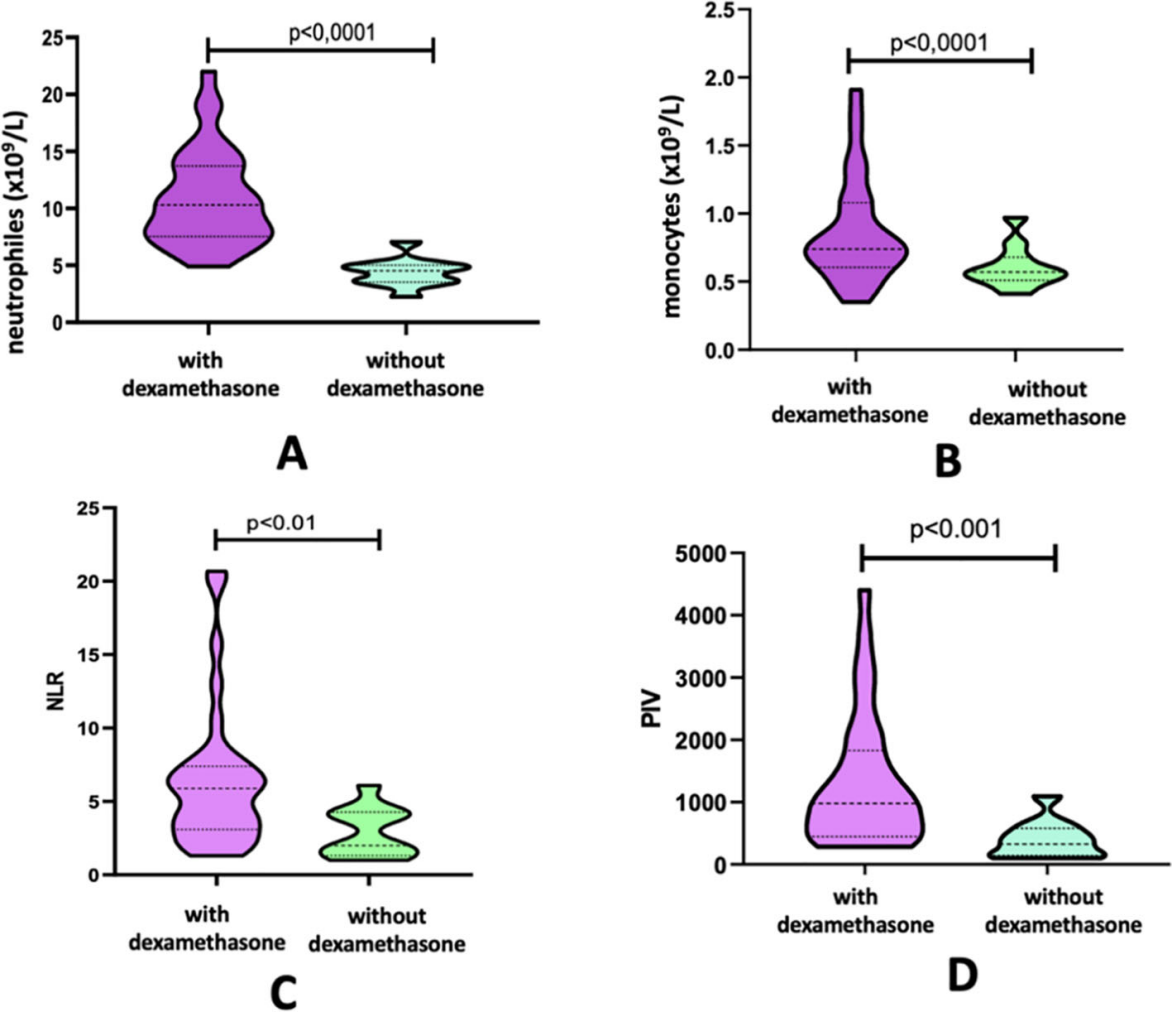
Absolute count of neutrophils **(A)**, monocytes **(B)**, and indices NLR **(C)** and PIV **(D)** in the Glioblastoma Group According to the Administration of Dexamethasone.

In the group with glioblastoma, the administration of dexamethasone did not affect the indices of lymphocytes, platelets, as well as the level of LMR, PLR. Absolute indices of neutrophils [10.3 (7.56-13.4)] and monocytes [0.74 (0.61-1.06)], the levels of NLR [5.88 (3.2-7.2)] and PIV [981 (450.25-1647.7]) were significantly higher than in the subgroup of patients who were not prescribed dexamethasone [4.54 (3.6-4.92); 0.57 (0.52-0.65); 2 (1.55-4.19); 328.6 (150.7-527.2)] (**Figure 2**).

**Figure 3:**
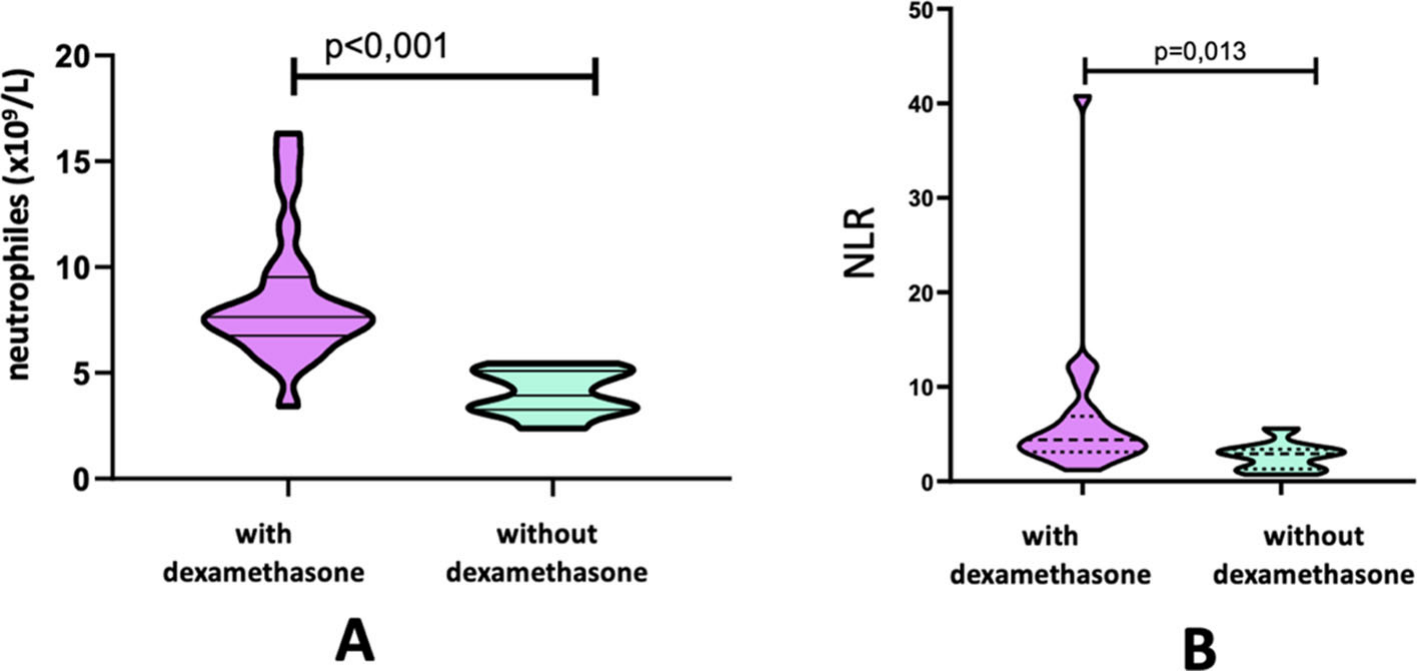
Absolute count of neutrophils **(A)** and indices NLR **(B)** in the group of patients with brain metastases according to the administration of dexamethasone.

In patients with metastatic CNS lesions, the administration of GCS had a significant effect only on the neutrophil level (7.64 [6.89-9.39] vs 3.93 [3.29-5.08]) and the NLR index (4.4 [3.15-6.5] vs 2.93 [133-333]) (**Table 4**) (**Figure 3**).

No statistically significant differences were observed in lymphocyte, monocyte, or platelet counts, nor in the LMR, PLR, or PIV indices, between patients receiving dexamethasone and those not receiving this therapy.

### 3.4 Diagnostic value of inflammatory blood markers

**Figure 4:**
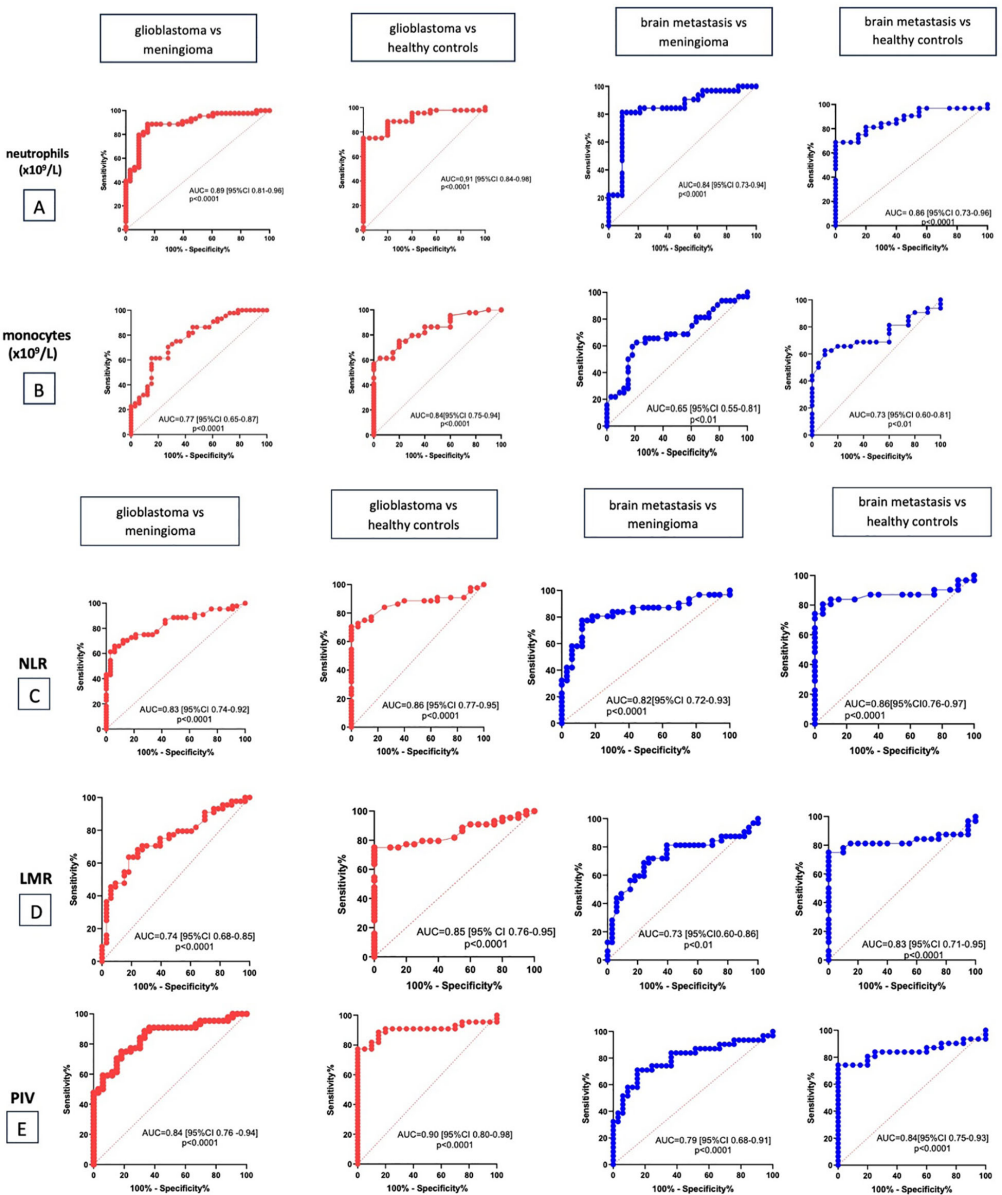
The diagnostic value of absolute count of neutrophils **(A)**, monocytes **(B)**, NLR **(C)**, LMR **(D)**, PIV **(E)**.

ROC analysis was used to confirm the diagnostic significance of the studied biomarkers. The results are presented in **Table 5** and **Figure 4**.

**Table 5:** Diagnostic value of various inflammatory markers.

Markers	Glioblastoma vs meningioma		Glioblastoma vs healthy controls		Brain metastasis vs meningioma		Brain metastasis vs healthy controls	
	AUC (95% Cl)	Cut off value	AUC (95% Cl)	Cut off value	AUC (95% Cl)	Cut off value	AUC (95% Cl)	Cut off value
neutrophils (x10^9^/L)	0.89	4.98	0.91	4.53	0.84	4.99	0.86	5.075
monocytes (x10^9^/L)	0.77	0.59	0.84	0.56	0.65	0.63	0.73	0.55
NLR	0.83	2.35	0.86	1.95	0.82	2.5	0.86	1.95
LMR	0.74	3.12	0.85	4.15	0.73	3.09	0.83	3.9
PIV	0.84	359.8	0.9	273.2	0.79	361.2	0.84	236.5

The optimal ratio of sensitivity and specificity for the diagnosis of glioblastoma compared to meningioma was observed for the count of neutrophils > 4.98 (79.55% and 90.91%, respectively), the count of monocytes > 0.59 (70.45% and 72.73%, respectively), NLR > 2.35 (75% and 78.79%, respectively), LMR < 3.12 (68.18% and 75.76%, respectively) and PIV > 359.8 (77.27% and 75.76%, respectively). When differentiating the diagnosis of glioblastoma from healthy controls, the optimal ratio of sensitivity and specificity was observed for the count of neutrophils > 4.53 (88% and 80%, respectively), the count of monocytes > 0.56 (75% and 80%, respectively), NLR > 1.95 (84% and 75%, respectively), LMR < 4.15 (77.27% and 75%, respectively) and PIV > 273.2 (90.91% and 80%, respectively).

When comparing markers in group of brain metastasis with meningioma the optimal ratio of sensitivity and specificity was observed for the count of neutrophils > 4.99 (81.25% and 80.91%, respectively), the count of monocytes > 0.63 (62.5% and 78.79%, respectively), NLR > 2.5 (80.65% and 78.79%, respectively), LMR < 3.09 (68.75% and 75.76%, respectively) and PIV > 361.2 (74.19% and 75.76%, respectively).

The optimal ratio of sensitivity and specificity for the diagnosis of brain metastasis compared to healthy controls was observed for the count of neutrophils > 5.07 (78.13% and 80%, respectively), the count of monocytes > 0.55 (65.63% and 75%, respectively), NLR > 1.95 (83.87% and 75%, respectively), LMR < 3.9 (81.25% and 85%, respectively) and PIV > 236.5 (83.87% and 75%, respectively).

However, the count of neutrophils and monocytes, NLR and PIV values in group with glioblastoma and the count of neutrophils and NLR in group with brain metastasis were influenced by the administration of GCS (**Tables 3**, **4**). LMR was unaffected by this therapeutic intervention, establishing LMR as a more robust independent marker.

It should be noted that PIV and the count of monocytes demonstrated diagnostic significance in group with metastatic brain lesions. In this patient group, PIV levels and the count of monocytes were not affected by dexamethasone administration (**Table 4**).

### 3.5 Analysis of preoperative inflammatory markers in CNS tumor patients not receiving dexamethasone

Among the study cohort, 11 patients with glioblastoma, 8 with brain metastases, and 29 with meningioma did not receive preoperative dexamethasone. Laboratory parameters and inflammatory biomarker levels for these subgroups are presented in **Table 6**.

**Table 6:** Preoperative laboratory parameters and assessed indices in three patient groups without dexamethasone, Me [Q1-Q3].

Parameters	Group 1 patients with glioblastoma (n = 11)	Group 2 patients with brain metastases (n = 8)	Group 3 patients with meningioma (n = 29)
lymphocytes (x10^9^/L)	1.26 [1.16-2.60]	1.57 [1.30-2.22]	2.03 [1.38-230]
neutrophils (x10^9^/L)	**4.54** **[3.60-4.92]** **α**	3, 93 [3.29-5.08]	**3.41** **[2.70-4.01]**
monocytes (x10^9^/L)	0.57 [0.52-0.65]	0.67 [0.41-0.70]	0.48 [0.37-0.56]
platelets (x10^9^/L)	218.00 [195.00-268.5]	226.50 [191.00-306.50]	236 [204 - 299]
albumin (g/l)	41.84 [41.09-44.19]	42.67 [39.25-45.89]	43.39 [41-44.54]
NLR	**2** **[1.55-4.19]** **β**	2.93 [1.33-3.33]	**1.57** **[1.2-2.2]**
LMR	**2.80** **[1.97-4.14]** **β**	2.9 [1.89-5.78]	**4.2** **[3.14-5.5]**
PLR	139 [82.5-186.5]	174 [84-255]	125 [103-162]
PIV	328.66 [150.7-527.2]	**358.45** **[181.36-764.29]** **β**	**167.84** **[127.86-314.86]**

α p<0.05 vs meningioma.

β p=0.06-0.08 vs meningioma.

NLR, neutrophil-to-lymphocyte ratio; LMR, lymphocyte-to-monocyte ratio; PLR, platelet-to-lymphocyte ratio; PIV, pan-immune-inflammation value.

**Figure 5:**
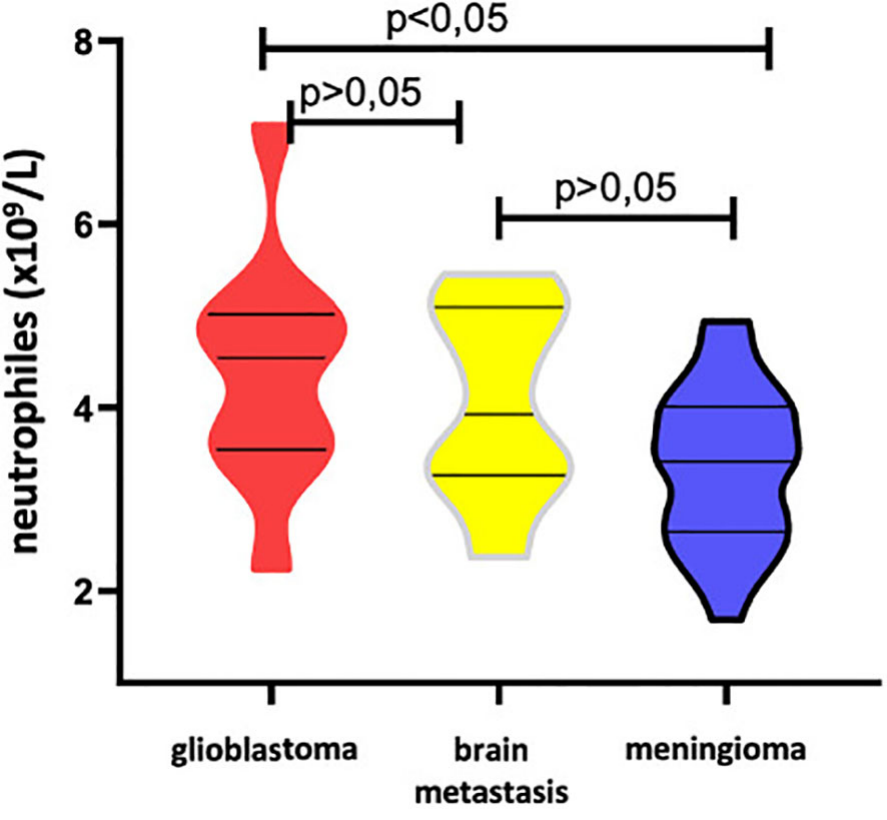
Absolute count of neutrophils in the group of patients with in glioblastoma, brain metastases, meningioma without dexamethasone.

A statistically significant difference was found only in the absolute neutrophil count between the glioblastoma and meningioma patient groups [4.54 vs. 3.41, p < 0.05] (**Figure 5**). Trends toward statistical significance were observed for higher NLR and lower LMR levels in glioblastoma patients compared to those with meningioma [2.0 vs. 1.57, p = 0.06; 2.8 vs. 4.2, p = 0.08].

A comparison of PIV levels among the study groups revealed a difference at the threshold of statistical significance between patients with brain metastases and those with meningioma [358.45 vs. 167.84, p=0.007].

No significant differences were observed between the groups in terms of absolute lymphocyte, monocyte, platelet counts and the PLR indices.

## 4 Discussion

Research has established that tumors are not merely clusters of mutated cells but complex ecosystems that integrate with and interact with the patient’s body. Tumor-associated inflammation is a key driver of oncogenesis, primarily mediated by immune cells (1, 2). Tumors recruit immune cells into their microenvironment and reprogram them to support oncogenesis and suppress immune responses. For instance, tumor-associated neutrophils alter the microenvironment by secreting angiogenic growth factors and matrix metalloproteinases while inhibiting the cytotoxic activity of T cells and natural killer cells (29). Similarly, tumor-associated macrophages express various immunomodulatory molecules that help suppress apoptosis, maintain a pro-inflammatory state, and facilitate tumor cell migration (14, 19, 30). Myeloid-derived suppressor cells further dampen anti-tumor immunity by impairing the function of effector T lymphocytes through metabolic regulation within the tumor microenvironment (31).

This chronic inflammatory state at the systemic level leads to measurable changes in circulating immune cells – neutrophils, lymphocytes, monocytes, and platelets (19). Insights from fundamental research have successfully translated into clinical practice. Current evidence highlights the significant diagnostic and prognostic utility of systemic inflammation biomarkers – such as NLR, LMR, PLR, and prognostic nutritional index (PNI) – in patients with extracranial solid tumors (6–11, 32). Elevated NLR, PLR, and PNI are consistently associated with poorer prognosis, whereas a higher LMR is generally a favorable prognostic marker.

For a long time, the CNS was considered an immune-privileged site, isolated from systemic immunity. However, it is now understood as an immunologically active environment. This is evidenced by the phagocytic and antigen-presenting functions of microglia, a network of meningeal lymphatic vessels connected to the peripheral immune system, and the capacity for lymphocyte infiltration into the brain parenchyma during pathology (33–36). Consequently, inflammatory responses within the CNS exhibit distinct features and constraints. These arise, first, from the high risk of collateral damage from an active immune response in such a vital organ, and second, from the unique nature of the pathological lesion itself (18).

Research into the immunological landscape of brain tumors has revealed specific patterns in the composition and diversity of tumor-infiltrating immune cells (37–39). It has been established that the abundance and phenotype of these immune cells correlate with the tumor’s dominant genetic alterations (37, 38). For instance, mesenchymal glioblastoma, which carries an extremely poor prognosis, is characterized by pronounced infiltration of tumor-associated macrophages, neutrophils, and regulatory T cells (Tregs), all exerting potent immunosuppressive effects (40). In contrast, astrocyte-like (or classical) glioblastomas exhibit limited immune infiltration, predominantly by macrophages. Proneural glioblastomas show the lowest levels of immune cell infiltration. Given the well-documented intratumoral heterogeneity of glioblastoma – both morphological and molecular – these correlations should be interpreted as general trends rather than absolute rules.

Other gliomas harboring IDH1/2 mutations, which are associated with a more favorable prognosis than glioblastoma, exhibit limited immune cell infiltration. The predominant populations in their microenvironment are anti-tumor M1 macrophages and activated cytotoxic T lymphocytes (CD8^+^ T cells) (38).

Investigations of the immune landscape in meningiomas have revealed an inverse correlation between immune cell infiltration density and tumor grade. Notably, the tumor-infiltrating lymphocytes (TILs) are predominantly cytotoxic CD8^+^ T cells, while immunosuppressive regulatory T cells (Tregs) are scarce (41). These findings underscore the significant role of the local immune response in disease progression and prognosis.

In metastatic CNS lesions, the degree and phenotype of immune infiltration are largely determined by the histogenesis (cell of origin) of the primary tumor. The highest density of anti-tumor cytotoxic T lymphocytes within CNS metastases is observed in so-called “immunologically hot” tumors, such as melanoma (42, 43). Unsurprisingly, immune checkpoint inhibitor therapy has shown considerable efficacy against such metastases in clinical studies (42). In contrast, brain metastases from breast cancer are often characterized by pronounced infiltration with tumor-associated macrophages (TAMs), and immunotherapeutic approaches have generally not demonstrated significant efficacy for these lesions in clinical trials (44).

The investigation of systemic immune responses in brain tumors is a relatively recent field of inquiry. It has been established that patients with malignant gliomas exhibit a significant reduction in circulating T-lymphocyte levels, attributed to their sequestration in the bone marrow and thymic involution (38, 45, 46). This lymphopenia may stem from the suppression of acute inflammation in a vital organ, although a direct influence of the tumor itself cannot be ruled out. In line with these findings, our results showed no statistically significant association between blood lymphocyte levels and tumor histological subtype, either between patient groups or when compared with healthy volunteers (p > 0.05). It is important to note that our analysis did not differentiate lymphocyte subpopulations.

The predominant cellular component in the microenvironment of malignant gliomas is tumor-associated macrophages (TAMs) (40), which originate from both resident microglia and peripheral monocytes. Consequently, alterations in peripheral blood monocyte counts in these patients are expected. In our study, the highest monocyte count was observed in the glioblastoma group (0.72 [0.55–0.94] x 10^9^/L). This value was significantly higher than in both the meningioma group (0.72 vs. 0.49, p < 0.05) and the control group (0.72 vs. 0.48, p < 0.05). For patients with metastatic brain lesions, a statistically significant elevation in monocyte count was noted in comparison to healthy controls and meningioma group (0.66 vs. 0.48, p < 0.05; 0.66 vs 0.49, p<0.05).

The LMR is a composite index reflecting the balance between adaptive immunity (lymphocytes) and innate myeloid lineage activity (monocytes). In oncology, this biomarker is widely used for diagnosis, prognosis assessment, and predicting response to immunotherapy (6–10). However, its clinical significance in neuro-oncology remains incompletely defined. Several studies support the diagnostic and prognostic value of LMR in glioma patients (27, 47, 48), with higher LMR levels associated with better outcomes and more frequently observed in low-grade gliomas (49). In our study, the lowest LMR values were recorded in the glioblastoma group (2.60 [1.71–3.91]) and patients with metastatic brain lesions (2.6 [1.88-3.77]). These values were significantly lower than those in both the meningioma group (2.6 vs. 4.2, p < 0.05) and the healthy control group (2.6 vs. 4.55, p < 0.05). The low LMR is likely attributable to the high absolute monocyte count observed in this cohort.

Neutrophils are the first inflammatory cells to infiltrate a pathological site. It is established that CNS tumors, including gliomas, express various cytokines to recruit and reprogram these cells to support tumor growth. Similar mechanisms are implicated in the formation of pre-metastatic niches and the development of CNS metastases.

Neutrophilia in patients with malignant gliomas has been documented in several studies (26, 48, 50, 51). Consistent with these reports, our data show that neutrophil counts were significantly higher in patients with glioblastoma and CNS metastases compared to both meningioma patients (8.36 vs. 3.54, p < 0.05; 7.05 vs. 3.54, p < 0.05) and healthy volunteers (8.36 vs. 3.39, p < 0.05; 7.05 vs. 3.39, p < 0.05).

In oncology, NLR and PIV are key prognostic biomarkers. Their clinical relevance extends to CNS tumors (26, 29, 48, 50, 52). Patients with high-grade gliomas and brain metastases exhibit significantly higher levels of these indices compared to those with benign extracerebral tumors. A positive correlation has also been demonstrated between glioma malignancy grade and NLR, PIV values (49, 51). These findings are expected, given the pronounced neutrophilia observed in glioblastoma and metastatic disease. Our results align with this body of evidence. Both NLR and PIV were significantly elevated in the glioblastoma and metastasis groups relative to the meningioma group (NLR: 4.29 vs. 1.60, p < 0.05; PIV: 759.5 vs. 180.22, p < 0.05) and healthy controls (NLR: 4.29 vs. 1.70, p < 0.05; PIV: 759.5 vs. 187.0, p < 0.05).

Thrombocytosis has long been associated with poor prognosis in oncology (15, 53). However, its role is currently being reevaluated in conjunction with other markers of systemic inflammation. One such significant integrative parameter is the PLR — the ratio of absolute platelet count to lymphocyte count — whose diagnostic and prognostic value has already been confirmed in non-small cell lung cancer, salivary gland carcinoma, oral squamous cell carcinoma, urothelial carcinoma and other malignancies (6, 7, 10, 11, 53). Several studies have demonstrated the prognostic significance of PLR in patients with glioblastomas (23, 26, 28). However, its diagnostic utility in neuro-oncology remains a subject of debate (24). Our data did not reveal statistically significant differences in PLR levels among the study groups (p ≥ 0.05).

A critical limitation of many prior studies is their failure to account for GCS use. Dexamethasone, introduced into neuro-oncology in the 1970s, remains the most frequently prescribed drug in this class (20). Its primary benefit for brain tumor patients is the restoration of blood-brain barrier integrity and reduction of peritumoral edema, leading to transient symptomatic relief. It is most commonly administered to patients with malignant gliomas and brain metastases.

However, GCS have broad systemic effects. Dexamethasone administered perioperatively is known to significantly compromise immune function (20, 54). It suppresses the proliferation and differentiation of naïve T-lymphocytes, thereby inhibiting early immune responses and reducing tumor infiltration by immune effectors (20, 39, 55). The observed increase in neutrophil and monocyte counts in patients on dexamethasone is attributed to the release of immature cells from the bone marrow and reduced expression of endothelial adhesion molecules. This results in the sequestration of these immune cells in the circulation, impeding their migration into tissues. It is important to note that the effect of dexamethasone varies with both the dose and the duration of therapy.

In our cohort, dexamethasone was administered to reduce peritumoral edema and alleviate neurological symptoms in 33 (75%) glioblastoma patients, 24 (75%) patients with brain metastases, and 4 (12%) patients with meningioma. A comparative analysis revealed that dexamethasone administration significantly influenced several cellular inflammatory markers in patients with glioblastoma and brain metastases.

In glioblastoma patients receiving GCS, neutrophil and monocyte counts, as well as NLR and PIV values, were significantly higher than in those not receiving treatment (neutrophils: 10.3 vs. 4.54, p < 0.05; monocytes: 0.74 vs. 0.57, p < 0.05; NLR: 5.88 vs. 2.00, p < 0.05; PIV: 981 vs 328.66),. In contrast, for patients with metastatic lesions, dexamethasone significantly increased only neutrophil count (7.64 vs. 3.93, p < 0.05) and NLR (4.40 vs. 2.93, p < 0.05). Notably, a dexamethasone-associated increase in monocyte count was observed in glioblastoma but not in the metastasis group. This discrepancy likely reflects distinct systemic immune responses to GCS in these tumor types and warrants further investigation. Importantly, dexamethasone did not affect the LMR, which retained its diagnostic significance in glioblastoma patients (p > 0.05). A significant limitation of our study pertains to the duration and dosage of therapy. The study included patients who received dexamethasone for a period ranging from 1 to 3 weeks, with a daily dose varying between 8 and 12 mg. It is important to note that the use of different dexamethasone doses or alternative dosing regimens may alter biomarker levels and the proposed cut-off values. However, the overall patterns and relative differences between the tumor groups identified in our study are likely to remain consistent.

Given the impact of GCS on the immune system and the obtained results, we assessed inflammatory markers in patients not receiving dexamethasone. Although no statistically significant differences were recorded, it is noteworthy that the lowest lymphocyte count was observed in patients with glioblastoma (1.26 vs. 1.57 vs. 2.03). A trend toward statistical significance was observed for higher NLR and lower LMR levels in the glioblastoma group compared to the meningioma group (2.0 vs. 1.57, p = 0.06; 2.8 vs. 4.2, p = 0.07). A difference in PIV approaching statistical significance was found between patients with metastatic brain lesions and those with meningioma (358.45 vs. 167.84, p = 0.07). The only parameter showing a statistically significant difference was the absolute neutrophil count, which was higher in the glioblastoma group than in the meningioma group (4.54 vs. 3.41, p < 0.05). It is important to note the relatively small sample sizes in the glioblastoma (n = 11) and CNS metastasis (n = 8) groups. The results of this subgroup analysis are exploratory and suggest that the diagnostic value of the investigated biomarkers may be independent of GCS therapy. Further large-scale, targeted studies are required to confirm this hypothesis.

No significant differences in inflammatory biomarker levels were found between patients with meningiomas (grades 1 and 2) and healthy volunteers (p > 0.05). This aligns with the typically indolent and non-aggressive clinical course of these lower-grade tumors, which are characterized by slow growth and a favorable prognosis.

Significant differences were observed in inflammatory biomarker profiles between patients with malignant intracerebral tumors (glioblastoma, metastases) and groups with meningioma and healthy volunteers in our study. In contrast, inflammatory profiles did not differ significantly between the glioblastoma and brain metastasis cohorts. This lack of difference likely reflects their shared aggressive biological behavior, marked by high proliferative activity and rapid growth.

However, it should be emphasized that the diagnostic significance of specific biomarkers varied between groups with glioblastoma and CNS metastases versus patients with meningioma and healthy controls. Specifically, the diagnostic value of monocyte counts and the PIV in glioblastoma patients was confounded by the influence of dexamethasone on these parameters. In contrast, in patients with metastatic disease, these same biomarkers were independent of glucocorticoid administration and remained significantly different from the levels observed in both meningioma patients and healthy volunteers.

It is important to note that, in line with international studies, our cohort of patients with metastatic disease was heterogeneous in terms of the primary tumor’s biological characteristics. It is established that certain cancers, such as non-small cell lung cancer, melanoma, and renal cell carcinoma, elicit a robust immune response. Conversely, other malignancies, including breast cancer or cancers of the gastrointestinal tract, are considered immunologically “cold” tumors, characterized by low immune cell infiltration and a weak immune response. A common feature of these diverse tumors is their tropism for the CNS and their ability to form brain metastases. In our study, the group with brain metastasis specifically comprised patients with disease progression confined solely to the CNS. Future research should aim to investigate these phenomena with stratification based on the biological subtype of the primary tumor.

This study has several important limitations that should be considered when interpreting the findings. First, the spectrum of tumor entities was limited. Only patients with glioblastoma, brain metastases, and meningioma were included, while other clinically relevant CNS neoplasms, such as lower-grade gliomas, primary CNS lymphomas, and ependymal tumors, were not analyzed. This restricts the generalizability of the results across the broader landscape of neuro-oncological diseases. Second, the overall sample size was relatively modest, particularly within key subgroups. Although the total cohort included 44 patients with glioblastoma, 32 with brain metastases, and 33 with meningioma, the number of patients not exposed to GCS was small (glioblastoma: n = 11, 25%; CNS metastases: n = 8, 25%; meningioma: n = 4, 12%). This may have limited statistical power, increased the risk of type II error, and reduced the robustness of subgroup-level inferences. Third, the metastatic cohort was biologically heterogeneous with respect to primary tumor origin. Differences in systemic inflammatory patterns across primary tumor types may have confounded the interpretation of inflammatory indices and contributed to variability in biomarker performance. Due to the limited cohort size, we did not perform stratification into immunologically “hot” and “cold” tumors, which constitutes a limitation of our study.

Fourth, the study focused exclusively on peripheral blood–based inflammatory markers and did not incorporate direct assessment of the tumor immune microenvironment. The absence of histological or molecular characterization of immune cell infiltration within tumor tissues prevented correlation of systemic inflammatory indices with local immune contexture. Fifth, there is a limitation in the evaluation of the effect of dexamethasone in the study. The study included patients receiving dexamethasone for a period of between 1 and 3 weeks. The dexamethasone dose ranged from 8 to 12 mg. Given the small number of patients, no stratification into subgroups based on dose or duration of therapy was performed. Finally, the study was designed primarily to explore diagnostic potential, and therefore did not evaluate prognostic or predictive associations. The lack of survival analyses, treatment–response correlations, and longitudinal follow-up limits the ability to determine the clinical utility of these markers for risk stratification or therapeutic decision-making.

Future studies should include larger, multicenter cohorts, expand the spectrum of tumor entities, incorporate standardized steroid exposure protocols, and integrate peripheral and tissue-based immune profiling to better define the biological and clinical relevance of cellular inflammatory biomarkers in CNS tumors.

## 5 Conclusion

**Figure 6:**
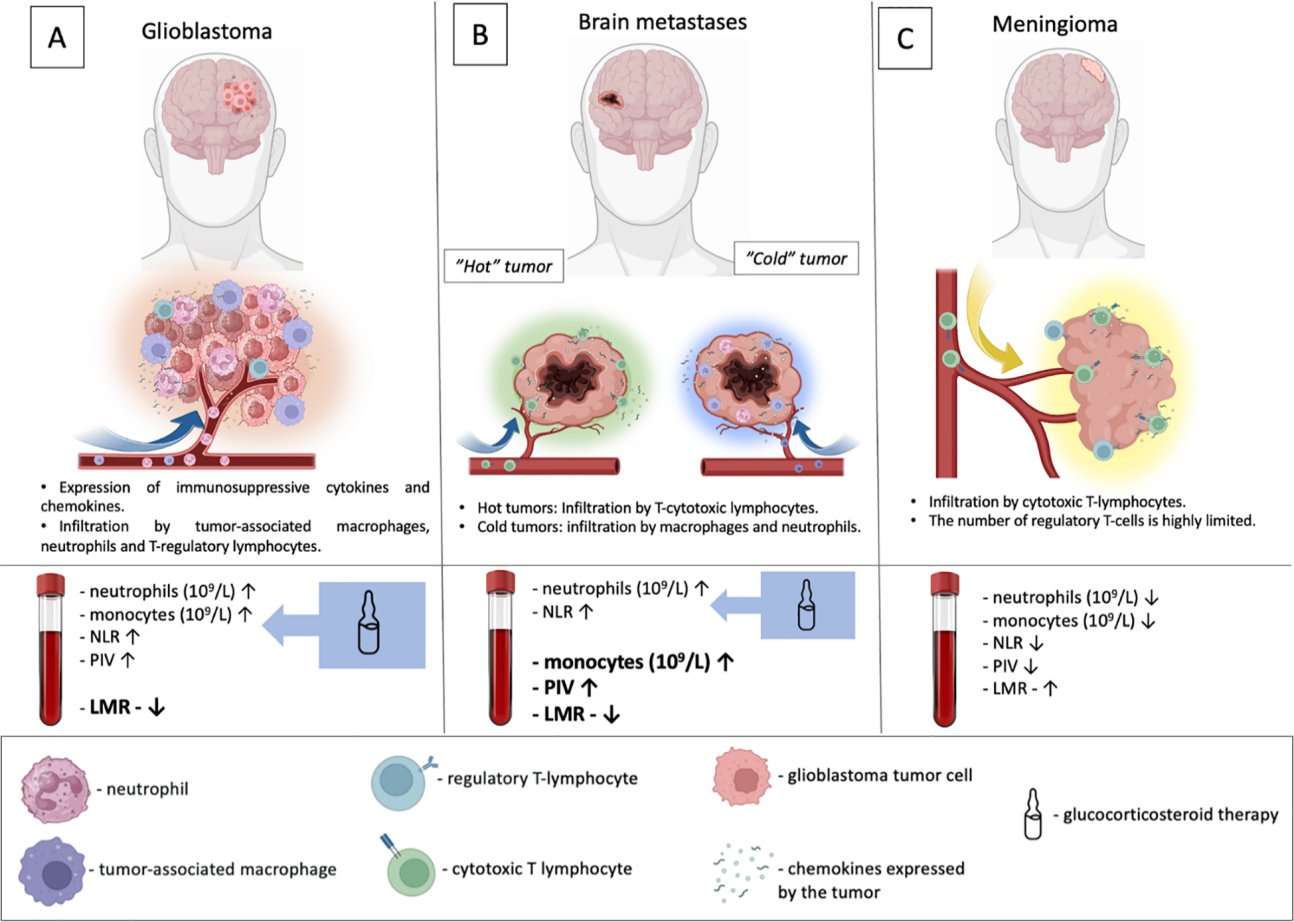
Local and systemic immune-inflammatory responses in CNS tumors. **(A)** Glioblastomas are characterized by establishing a profoundly immunosuppressive microenvironment, heavily infiltrated by tumor-associated macrophages, neutrophils, and T-regulatory lymphocytes that inhibit an effective immune response. Systemically, these patients displayed elevated levels of neutrophils, monocytes, NLR, PIV, along with a decreased LMR compared to patients with meningioma and healthy volunteers. Dexamethasone administration significantly affected neutrophil and monocyte counts, as well as NLR and PIV values. In contrast, LMR levels were independent of dexamethasone treatment. **(B)** In CNS metastases, the primary tumor histogenesis is a key determinant of the immune-inflammatory response. Immunologically "hot" tumors are characterized by prominent infiltration with cytotoxic T cells. In contrast, immunologically "cold" neoplasms are predominantly populated by tumor-associated macrophages and neutrophils. Systemically, patients exhibited elevated levels of neutrophils, monocytes, PIV, NLR, alongside a decreased LMR. Notably, both LMR and PIV levels were independent of dexamethasone administration. **(C)** In meningiomas, infiltrating tumors were represented by cytotoxic lymphocytes. In blood parameters in patients with meningioma, a decrease in the level of neutrophils and monocytes, NLR, PIV, as well as an increase in LMR compared with patients with intracerebral tumors was recorded.

Local and systemic immune–inflammatory responses in CNS tumors are shaped both by intrinsic tumor biology and by the widespread use of GCS, which substantially influence commonly used hematological inflammatory indices (**Figure 6**). According to our results, significant differences in absolute neutrophil and monocyte counts, as well as in NLR, LMR, and PIV levels, were found between patients with intracerebral tumors (glioblastoma and brain metastases) and those with meningioma. However, in the glioblastoma group, the diagnostic utility of neutrophil and monocyte counts, NLR, and PIV is confounded by their elevation due to GCS therapy. In contrast, the LMR level was not influenced by GCS administration. In patients with metastatic brain lesions, dexamethasone administration resulted in a significant increase in absolute neutrophil count and NLR. In contrast, absolute monocyte count, LMR, and PIV demonstrated diagnostic significance irrespective of GCS therapy.

The primary diagnostic value identified in our study lies not in differentiating glioblastoma from brain metastases (where their inflammatory profiles are similar, reflecting a shared aggressive phenotype), but in distinguishing malignant intracerebral tumors from meningioma and/or the absence of disease.

In cases with ambiguous or multifocal presentation on magnetic resonance imaging (MRI), abnormally high PIV and low LMR values can heighten suspicion of a malignant process (glioblastoma or metastasis).

Furthermore, for a patient with a known oncological history and a new brain lesion, elevated absolute monocyte count and PIV, along with a low LMR – particularly if these values are unaffected by dexamethasone administration – can serve as an additional argument in favor of a metastatic rather than a primary glial origin. However, to validate these findings, further multicenter studies are required, accounting for variability in clinical practices, patient demographics, and employing standardized statistical analysis.

Collectively, these results highlight the potential of peripheral cellular inflammatory markers as accessible, low-cost tools to aid in the biological stratification of CNS tumors. Further mechanistic and prospective studies are warranted to clarify their role in clinical decision-making, improve patient risk stratification, and support the development of more personalized therapeutic strategies in neuro-oncology.

## Data Availability

The raw data supporting the conclusions of this article will be made available by the authors, without undue reservation.
